# Does non-visible haematuria require urgent assessment? A retrospective cohort study from a university teaching hospital

**DOI:** 10.1007/s00345-021-03670-0

**Published:** 2021-03-24

**Authors:** James Lucocq, Adnan Ali, William Harrison, Tarek Khalil, Gursunil Powar, Kamran Raza, Ghulam Nandwani

**Affiliations:** Department of Urology, Ninewells Hospital, Dundee, Scotland

**Keywords:** Microscopic haematuria, Urological cancer, Risk factors

## Abstract

**Objectives:**

It is not certain from current evidence which patient groups with non-visible haematuria (NVH) require urgent investigation and which investigations are sufficient. We report referral outcomes data from Scotland to identify patient groups who will benefit from urgent assessment to rule out urological cancer (UC) and whether full set of investigations are necessary in all referred patients.

**Materials and methods:**

Data were collected from electronic patient records for patients referred with NVH to secondary care urology services between July 2017 and May 2020. The correlations between risk factors and final diagnosis were assessed using categorical variables in a multivariate logistic regression analysis and using chi-squared models. Statistical analysis was performed using IBM SPSS data editor version 25.

**Results:**

Our study cohort comprised 525 patients (43.4% males; median age 66 years), in which UC was diagnosed in 25 patients (4.8%). Age > 60 years had sensitivity and NPV for UC of 92% and 99%, respectively. Univariate and multivariate analysis showed male sex, age ≥ 60 years and smoking were significant predictors of UC in patients with NVH (*p* < 0.05). There was no significant difference in UC in patients with history of LUTS, anticoagulation and previous UC.

**Conclusion:**

The risk of urologic cancer in NVH patients is significant and male gender, age ≥ 60 years and smoking are significant predictors of UC. Patients with risk factors of UC require complete assessment of both the upper and lower urinary tract; however, in the absence of risk factors, patients do not require urgent or complete assessment.

## Introduction

The prevalence of non-visible haematuria (NVH) in the general population is around 2.5% but variability is seen with gender and age groups [1, 2]. Various methods and definitions were used to detect NVH but in practical terms, urine dipstick testing is a feasible and cost-effective method particularly in general practice [3, 4]. The studies have confirmed accuracy of dipstick haematuria with quantitative red blood cell microscopy [3, 5]. NVH referrals to secondary care are based on dipstick testing which is the preferred method for assessment in general practice [4]. The main concern is to exclude urological cancer and a recent metanalysis reported that NVH evaluation detected, bladder cancer (BC), kidney cancer (KC) and upper tract urothelial cancer (UTUC) in 3.2%, 0.28% and 0.04% of cases, respectively [6]. In general, diagnostic yield of significant benign cause is in the range of 28–38% and up to 100% including all less significant causes, depending on the method of investigations and population [7–9].

Investigation of NVH includes checking renal functions, upper tract assessment with imaging and flexible cystoscopy, along with clinical examination. Appropriate assessment of NVH uses significant resources, carries a cost and is also a challenge on already stretched secondary care urology services in NHS system [4, 10]. There is no uniform guidance on assessment of NVH patient [3]. NICE guideline for NVH suggests an urgent referral in patients aged ≥ 60 with dysuria or a raised white cell count and non-urgent referral in aged ≥ 60 with no explainable cause for NVH [11].

It is not certain from current evidence which patient groups with NVH require urgent investigation and which investigations are sufficient. We report referral outcomes data from Scotland to identify patient groups who will benefit from urgent assessment to rule out urological cancer and whether full set of investigations are necessary in all patients referred with NVH.

## Materials and methods

Data were collected from electronic patient records for patients presenting with non-visible haematuria between July 2017 and May 2020. A search was made for all patients referred with non-visible haematuria.

NVH was defined when urine dipstick was positive for 3 + blood on 2 consecutive urine samples, 1–2 weeks apart in the absence of urinary tract infection (UTI). All patients with new onset NVH regardless of symptoms were included in the study. Patients with recurrent NVH, under 16 years and incomplete investigations were excluded.

Our NVH assessment protocol include clinical history (smoking, symptoms, previous urological history, co-morbidities, medications and family history), focussed urological examination including digital rectal examination (DRE) in males, renal functions, full blood count, ultrasound kidneys and bladder (USS KUB), urine dipstick (urine culture where appropriate) and flexible cystoscopy (FC). Per-vaginal examination was not routinely performed. USS KUB was omitted in patients who already had upper tract assessment (CT urogram, CT Kidneys and Bladder (CT KUB), MRI Kidneys, MR urogram or CT Abdomen & Pelvis) within 3 months of referral for NVH. For patients with no malignancy at initial investigation, all subsequent electronic referral or admission records (including urology and other specialties) and radiological investigations were reviewed for 9 months, to assess any missed urological cancers.

Asymptomatic patients with urine dipstick positive for suspected UTI proceeded to FC under antibiotic cover or deferred till further evaluation for UTI at the discretion of the assessing urologist. In patients with UTI based on symptoms regardless of a positive urine dipstick for leucocytes and nitrites at the time of assessment, FC was deferred till UTI was treated with antibiotics and negative urine culture obtained. Once treated for UTI, these patients were relisted for completion FC. In those treated who required treatment for UTI prior to completion FC, we did not look for NVH as these initially had NVH in absence of UTI as per above NVH definition.

Lower urinary tract symptoms (LUTS) were defined as presence of storage or voiding symptoms. If further assessment based on severity of symptoms was needed, these patients were further followed up in designated LUTS clinic where IPSS scores, QoL questions and flow studies were done. For statistical analysis, we only assessed presence or absence of LUTS based on the above definition.

If patient was continually smoking at the time of referral or stopped smoking ≤ 1 year, it is defined as smoker. However, if smoking cessation was ≥ 1 year, it was labelled as Ex-smoker. For statistical analysis smoking variable included smokers and Ex-smokers.

Patients were divided in groups based on diagnostic pathology, gender, age, symptoms, and smoking history. Statistical analysis was done using IBM SPSS data editor version 25. Fisher’s exact test and chi-squared tests were applied to analyse the relationships between categorical variables. Differences between continuous variables were assessed by ANOVA and Mann–Whitney *U* test (where appropriate). The correlations between risk factors and final diagnosis were assessed using categorical variables in a multivariate logistic regression analysis and using chi-squared models. Risk factors associated with all malignancy (prostate, bladder, urethral, kidney) and bladder malignancy alone were interpreted.

## Results

Our study cohort comprised 525 patients (43.4% males; median age 66 years) who were referred for the evaluation of NVH from primary care service. History of smoking was present in 247 (47%), LUTS in 227 (43.2%), anticoagulation in 142 (27%) and previous cancer in 42 (8%). USS was the commonest investigation in 371 (70.7%) while CT scan was performed in 109 (20.8%). Combination of USS and CT was required in 38 (7.2%) while 5 (1%) already had MRI abdomen due to non-urological reasons when referred for NVH. Benign prostate enlargement 67 (12.8%) was the most identified pathology where benign diagnosis was made (Table 1).

**Table 1:** Patient characteristics and summary of findings

Age years [median (min–max), IQR]	66 (22–93), 22	
	Frequency	Percent
Male/female	228/297	43.4/56.6
Smoking history		
Current smoker	105	20
Ex-smoker	142	27
Non smoker	278	53
Renal function		
eGFR ≥ 60	485	92.4
Not done	5	1
Co morbidities		
Hypertension	133	25.3
Ischaemic heart disease/atrial fibrillation	89	17
Diabetes mellitus	35	6.7
Chronic obstructive pulmonary disease	44	8.4
Chronic kidney disease (any stage)	39	7.4
Miscellaneous	156	29.7
Any comorbidity	415	79
Urological causes		
No urological cause found	317	60.4
Urological cancer	25	4.8
Benign prostatic enlargement	67	12.8
UTI	41	7.8
Urolithiasis (renal/ureter/bladder)	23	4.4
Bladder neck stenosis/urethral stricture	20	3.8
Miscellaneous	32	6.1
Total	525	100.0

Urological cancer (UC) was diagnosed in 25 patients (4.8%). Bladder cancer was the commonest cancer in 20 (3.8%) followed by renal and prostate cancer in 3 (0.6%) and 2 (0.4%), respectively. Median age was 77 years (IQR 15) in GU cancer patients while in patients with no cause or benign pathology median age was 65 years (IQR 22), *p* < 0.0001. ROC analysis of age (years) cutoff values to detect UC showed a cut of value of 60 years provided the highest combined sensitivity and specificity of 92% and 40.2%, respectively.

UC was diagnosed in 18 patients (7.3%) of 247 patients (47%) with history of smoking and no difference was demonstrated for UC between ex-smokers and current smokers (9.2% and 4.8%, *p* = 0.22), respectively. There was no significant difference in UC in patients with history of LUTS, anticoagulation, antiplatelet therapy and previous UC. On univariate and multivariate analysis male sex, age ≥ 60 years and smoking (current smoking or ex-smoking) were significant predictors of UC in patients with NVH, *p* < 0.05 (Tables 2, 3). The combined factors of male sex, age > 60 years and smoking have a sensitivity, specificity, PPV and NPV for UC of 48.0%, 83.3%, 24.5% and 97.4%, respectively. The presence of at least one of the variables (male sex, age > 60 years, smoking) has a sensitivity, specificity, PPV and NPV of 100%, 16.7%, 5.3%, 100%. The presence of at least two of the variables has a sensitivity, specificity, PPV and NVP of 80%, 45.6%, 6.4%, 98.0%.

**Table 2:** Univariate logistic regression analysis of clinical predictors for urological cancer in NVH patients

	Odds ratio	*p* value	CI (95%)	
Male sex	4.41	0.002	1.73	11.23
Age ≥ 60 (years)	7.73	0.006	1.8	33.15
Smoker or ex-smoker	3.04	0.014	1.25	7.41
LUTS	0.87	0.74	0.38	2
Antiplatelet and/or anticoagulation	1.8	0.14	0.81	4.24

**Table 3:** Multivariate logistic regression analysis of clinical predictors for urological cancer in NVH patients

	Odds ratio	*p* value	CI (95%)	
Male	3.13	0.019	1.21	8.14
Age ≥ 60 (years)	6.00	0.017	1.37	26.23
Smoker or ex-smoker	2.54	0.045	1.02	6.32

Benign urological pathology (BUP) and no urological cause (NUC) were found in 183 (35%) and 317 (60.4%). Median ages between BUP and NUC were 70 years (IQR 18) and 60 (IQR 22; *p* < 0.0001). In 215 patients ≤ 60 years, NUC, BUP and UC (Bladder and renal) were found in 73.5%, 25.6% and 0.9% (0.45% and 0.45%), respectively. In 49 patients ≤ 40 years of age, NUC, BUP and UC were found in 75.5%, 24.5% and 0%, respectively.

## Discussion

Although a cause for NVH be found in over 32%, significant causes are found in a much lower proportion of cases [7, 9]. The combined percentage of patients with either urological cancer or significant non-cancer causes, ranges from 0 to 16% [6, 12, 13]. Edwards et al. evaluated 4020 haematuria patients and found urological malignancy in 4.8% of patients in the NVH group, while Mishriki et al. reported 6% urological cancer yield on assessment of 292 asymptomatic NVH patients [13, 14]. Our study also reports a low rate of urological cancer (4.8%) in this patient cohort. Some studies, such as Khan et al. report an even lower rate of UC in NVH patients (1.6%) [15]. A possible explanation is that we considered 3 + urine dipstick as threshold for NVH assessment. Jung et al. report increasing incidence of urologic malignancy with increasing degree of NVH [16].

Sensitivity and specificity of USS versus CT for detection for renal tumours were 14.3% and 100% versus 89% and 99.6%, respectively [6, 17]. UTUC detection in patients with NVH is 0.042% in NVH patients. In view of low incidence and categorising patients with risk factors for further assessment with CTU, chances of missing an UTUC is very unlikely with initial assessment with USS. It picks up indirect signs and based on these, further CTU assessment can be planned to exclude significant upper tract pathology. For upper tract assessment in NVH, USS kidney is the investigation of choice at our institution. Ongoing review of the subsequent referrals and radiological imaging for 9 months of our cohort makes it unlikely that we missed UTUC.

Despite full assessment of non-visible haematuria with cystoscopy, upper tract imaging and renal functions, often no pathology is identified. Mishriki et al. reports that 44% of patients have no pathology. Our findings suggest that up to 60.4% of patients have no underlying pathology.

Our findings suggest that a significant number of patients have benign pathology (34.9%), the majority of which is not significant. We believe that the high percentage of benign prostate hypertrophy most likely did not contribute to the non-visible haematuria, albeit there were some cases of hyper vascular prostates. In view of the low likelihood of cancer, yet high chance of non-significant/no pathology, there is a need to identify risk groups where full urgent assessment will be more appropriate versus those where a limited assessment should be sufficient. This will utilise resources more efficiently and save cost.

Studies have shown patients < 50 years with NVH to have a very low incidence of urological malignancy [12–14, 18]. The present study found no urological cancers in the patients ≤ 40 years and 75.5% had normal assessment. There were 2 patients with urological cancer < 60 years, one had upper tract cancer and another had carcinoma in situ. This showed extremely low incidence of urological cancer (0.9%) in this age group and 73.4% had normal investigations. Other studies such as Murakami et al. report a significant difference between sex; 6.6% and 1.6% urological cancer detection in males and females, respectively. Although bladder cancer was found in 1.25%, the vast majority were males; while in females, renal cancer was common [12]. Our study detected urological cancer in 8.3% and 2% of males and females, respectively, consistent with the findings of Murakami et al.

A study from Denmark conducted by Elmussareh et al. suggested asymptomatic NVH should not be routinely evaluated due to the low incidence of UC in patients with NVH. From January 2016 they revised their guidelines and NVH is no longer routinely investigated. Retrospective analyses of asymptomatic NVH referrals in Denmark report that this revised guideline would result in 0.8% (11/1305) to 1.5% (10/687) missed benign and malignant urological tumours [10, 11]. Instead of disregarding the evaluation of NVH completely, we encourage clinicians to use patient factors to guide both the decision to investigate and the urgency of investigation.

In our institution, all NVH patients referred to the urology department are investigated with renal function, DRE (males), Ultrasound kidneys and bladder (USS KUB) and a flexible cystoscopy. There is a need to lessen the strain on secondary urology services by prioritising patients at high risk of harbouring urological malignancy and significant benign urological causes. The risk factors as identified in the present study can be used as the basis of patient prioritisation to prevent overwhelming utilisation of urology services. In the absence of risk factors, patients can safely be investigated non-urgently (> 2 weeks) or indeed undergo a limited assessment, depending on the availability of resources.

## Data Availability

Data available on request.
